# Single Ventricle With Hypoplastic Left Heart Syndrome in a 22-Year-Old Male: A Rare Case Report

**DOI:** 10.7759/cureus.30050

**Published:** 2022-10-08

**Authors:** Riya Sharma, Pallavi Yelne, Sourya Acharya

**Affiliations:** 1 Medicine, Jawaharlal Nehru Medical College, Datta Meghe Institute of Medical Sciences, Wardha, IND

**Keywords:** cyanotic congenital heart disease, hypoplastic pulmonary atresia, ductus dependent, transposition of great arteries, single ventricle

## Abstract

Uni-ventricle transposition of great arteries with ductus-dependent pulmonary circulations with hypoplastic pulmonary atresia (PA) represents rare cardiac malformations. We are presenting a unique case of a 22-year-old male who complained of dizziness since morning with numbness of the left lower and upper limbs. He also complained of palpitations on and off for two to three years with episodes of cyanosis on exertion. He gave a history of cough with frothy pink sputum at the same time. At birth, he was diagnosed to have cyanotic congenital heart disease (CCHD), for which he was prescribed a combination of ambrisentan 5 mg and tadalafil 20 mg and tablet aspirin 75mg along with some lifesaving modalities like proper hydration and phlebotomy as and when required. He was told to go for high-risk surgery like Blalock Taussig shunt or bidirectional Glenn. A Blalock Taussig shunt is a short tube only a few mm wide, which makes a path for blood to go from the arterial circulation to the lungs and bidirectional Glenn sends blood directly from the upper body to the lungs. Strict compliance with drug therapy is implied for the patient for a better outcome.

## Introduction

The study of congenital heart disease (CHD) depicts the survival of a person with the disease in a population and the screening of its progress over a long period. CHD is one of the familiar congenital disabilities. There are two types of CHD: cyanotic (tetralogy, transposition of great arteries, truncus arteriosus, hypoplastic left heart syndrome) and acyanotic (ventricular septic defect, atrial septal defect, patent ductus arteriosus, coarctation). Most of them are acyanotic, approximately 75%.

Recent experimentation has shown genes encoding the substance of nucleus modifiers, proteins related to cilia, and cell signalling pathways transduced by cilia play an essential role in CHD pathogenesis [1]. The occurrence of CHD in different analyses differs from about 4/1,000 to 50/1,000 nascency. The relative recurrence of different CHD forms differs from research to research [2]. Here we are discussing the case of a single ventricle with hypoplastic left heart syndrome (HLHS ). Uni ventricle with pulmonary atresia is a complex cyanotic CHD (CCHD). Single ventricle anomaly involves various cardiac malformations in which the ventricle is underdeveloped or the septum is not formed, which results in the merging of oxygenated and deoxygenated blood. Different single ventricular variations include HLHS, tricuspid atresia, Ebstein anomaly, and double outlet left ventricle.

## Case presentation

History

A 22-year-old male presented with complaints of dizziness since morning, followed by upper limb and lower limb numbness. It was not associated with falls or episodes of seizures. He also complained of four to five episodes of vomiting, which was non-projectile and not associated with headache and diplopia. There was no history of hematemesis. He gave a history of cough with frothy pink sputum for two months. He was also complaining of palpitations on and off since the same time. He used to have episodes of palpitations every one to two months, which subsided on rest. He also complained of exercise intolerance (New York Heart Association class two) and cyanosis on exertion.

**Figure 1:**
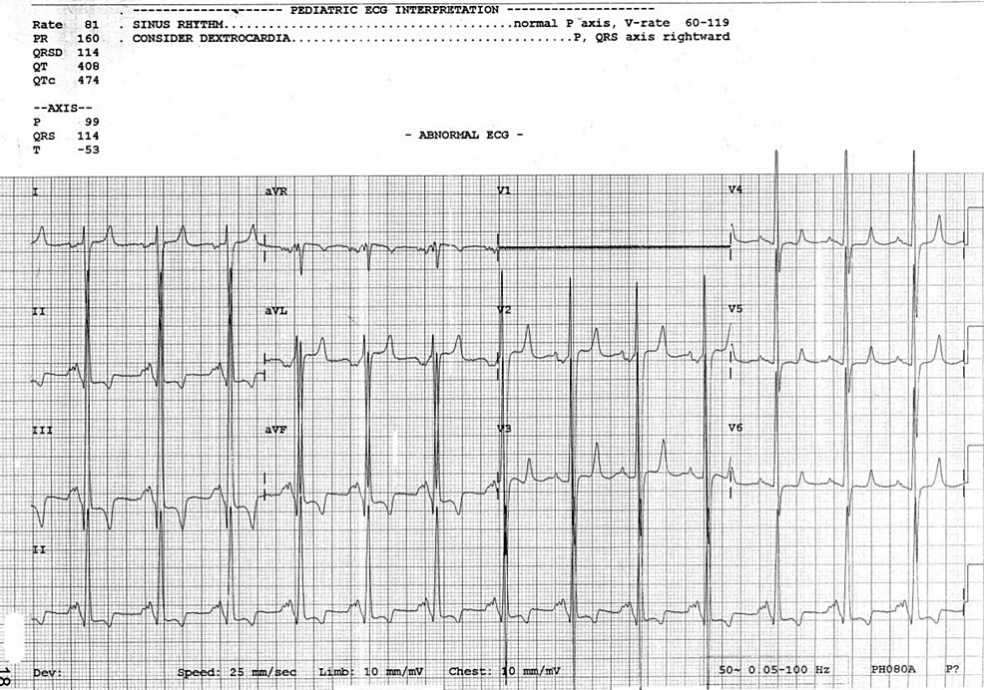
ECG showing single ventricle physiology ECG shows T wave inversion in inferior leads, biventricular hypertrophy, and left atrial overload.

On asking further, the mother gave a history of some congenital heart disease. On detailed questioning, she revealed that at birth, the child had respiratory distress and bluish discolouration of the face and extremities, for which he was given oxygen therapy but not relieved, so he was further investigated. The patient was having documents and investigation reports done at birth, which revealed that two-dimensional echocardiography (2D ECHO) Doppler was conducted, which was suggestive of complex cyanotic congenital heart disease that is single ventricle (morphologically right), single atrioventricular (AV) valve with single atrium, Transposition of great arteries with ductus dependent pulmonary circulations with hypoplastic pulmonary atresia, single AV valve shows cleft with valvular regurgitation as shown in Figure 1.

As it was a complex CCHD, he was referred to a higher cardiac centre and advised to undergo angiography for the correct assessment of CCHD, but due to some delay, it was done when he was one year old. Angiography was done, and it was suggestive of CCHD single ventricle and single atrioventricular valve with pulmonary atresia, ostium primum (OP) atrial septal defect with bidirectional shunt, and good ventricular function. He was then put on tablet propranolol 2.5 mg twice a day and syrup tonoferon, and was told to go for high-risk surgery like BT shunt (Blalock Taussig shunt) or BD Glenn (bidirectional Glenn). A Blalock Taussig shunt is a short tube only a few mm wide that makes a path for blood to go from the arterial circulation to the lungs, and BD GLENN sends blood directly from the upper body to the lungs. He was also asked to take medication for infective endocarditis prophylaxis.

**Figure 2:**
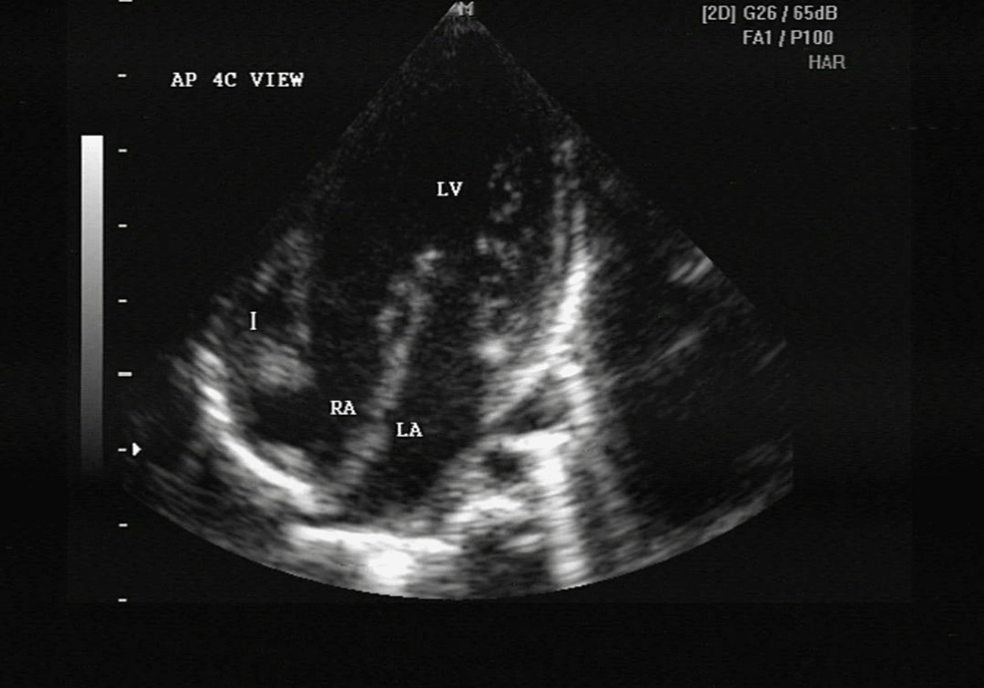
A 4-chambered view with rudimentary right ventricle (single ventricle-left ventricle) LV - left ventricle; LA - left atrium; RA - right atrium; I - cursor

Until 2014, he was normal except for occasional cough, cold, and discomfort due to exertion. In 2016 he complained of minor chest pain on the right side with uneasiness and was admitted, where he was managed with IV fluids and beta blockers. He was referred to a higher centre for complete diagnostic imaging, including CT/MR angiography, which revealed situs inversus, interrupted inferior vena cava, atrioventricular discordance, complete atrioventricular septal defect (AVSD) with left AV valve atresia, hypoplastic left ventricle, single ventricle (morphologically right) as shown in Figure 2. It is due to obstructive pulmonary vascular disease that there has been a development of pulmonary arterial hypertension. So, he was not fit for staged single ventricle palliation and was managed conservatively. Currently, he is maintaining proper hydration along with tablet pulmonext kit twice a day, tablet concor 2.5 mg once a day, and syrup tonoferon 5 ml daily. He also has undergone phlebotomy for raised haemoglobin.

Investigation

The patient was alert, responsive, and well-oriented to time, place, and person. On examination, the following were found: the patient was afebrile, pulse- 83/minute, blood pressure - 100/60 mm Hg, respiratory rate- 20/minute, oxygen saturation- 96%, hyperemia present in lower palpebral conjunctiva, peripheral and central cyanosis was present, clubbing (grade three) was present.

On cardiovascular system examination, the following were found: S1 - normal; S2 - single grade three ejection systolic murmur at the left parasternal border was heard. The bilateral vesicular breath sounds were heard, and crepitation was heard bilaterally in the lower zone on respiratory examination. On central nervous system examination, the patient had no neurological deficit. Bilateral planters were flexors. On examining the abdomen, soft, non-tender bowel sounds were present. On investigation, the following were found: haemoglobin - 22.7 g/dl, total leucocyte count- 7300/cu mm, platelets- 1.00 lacs/cu mm, prothrombin time - 25.2 sec, urea - 19 mg/dL, sodium - 134 mmol/L, potassium - 4.7 mmol/L, creatinine - 0.8 mg/dl, chloride - 100 mmol/L.

Treatment

The cardiologist's opinion was taken and managed accordingly. The doctor managed the patient with antiemetics injection ondansetron 4 mg thrice a day, intravenous fluids, antibiotics, injection pantoprazole, nebulization with Duolin, tablet Ecosprin AV (75+10 mg) at bedtime, tablet Homocheck once a day, tablet Pulmonext twice a day, tablet bisoprolol 2.5 mg once a day. Phlebotomy was done for secondary polycythemia. He was instructed to take proper hydration to prevent dehydration. He was asked to take urgent hospital care when symptoms like chest pain, breathlessness, sweat, or loss of consciousness were there.

## Discussion

Univentricular physiology with pulmonary atresia is a complex CCHD. Single ventricle anomaly involves various cardiac malformations in which the ventricle is underdeveloped or the septum is not formed, which results in the merging of oxygenated and deoxygenated blood. Different single ventricular variations include HLHS, tricuspid atresia, Ebstein anomaly, and double outlet left ventricle. Single ventricle could be identified earliest at the eighteenth week of gestation [3]. Congenital atresia is a rare asymptomatic condition if only one or two parts of the lungs are involved. It is lethal if it is associated with cardiac anomalies. In the latter case, they usually present with recurrent lung infections, blood in cough, dizziness, and cyanosis. Treatment of such patients is controversial and has a poor prognosis [4]. Presently interventional and therapeutic regimens for single ventricle anomalies remain beneficial. In the long run, in spite of treatment, the systemic ventricle has a great probability of advancing non-success. Development in stem cell therapy has been hopeful for the cure of heart failure. Various clinical experiments have been conducted to examine the therapeutic capacity that stem cell therapy may hold for pediatric inhabitants with single ventricle anomalies [5]. The single ventricle reconstruction experimentation has matured and has progressively shifted judgment away from the right ventricle shunt as a cause of upgrade results [6].

## Conclusions

Single ventricle patients require staged reconstruction. My patient was not fit for Glenn shunt, Fontan procedure, and Blalock Taussig shunt, so he was managed with tablet Ecosprin and tablet Homocheck. Phlebotomy was also done. Development in stem cell therapy has been hopeful for the cure of heart failure. 
